# Mix proportion and microscopic characterization of coal-based solid waste backfill material based on response surface methodology and multi-objective decision-making

**DOI:** 10.1038/s41598-024-56028-y

**Published:** 2024-03-07

**Authors:** Xinyuan Zhao, Ke Yang, Xiang He, Zhen Wei, Jiqiang Zhang, Xiang Yu

**Affiliations:** 1 State Key Laboratory of Mining Response and Disaster Prevention and Control in Deep Coal Mine, Anhui University of Science and Technology, Huainan, 232001 China; 2 Institute of Energy, Hefei Comprehensive National Science Center, Hefei, 230031 Anhui China

**Keywords:** Response surface methodology, Multi-objective decision-making method, Coal-based solid waste, Underground backfilling, Mix proportion, Engineering, Materials science

## Abstract

The mix proportion of multi-source coal-based solid waste (CSW) for underground backfilling affects transportation and support performance of backfill materials, and even the backfilling cost. In this study, the optimal mix proportion of desulfurization gypsum (DG), furnace bottom slag (FBS) and gasification fine slag (GFS) is determined by the Response Surface Methodology–Box Behnken Design (RSM-BBD). Then the fluidity, bleeding rate, 3-day strength, 7-day strength and preparation cost are evaluation indicators, the optimal mix proportion of backfill materials is determined by the multi-objective decision-making method (MDM). Finally, the microstructure of the backfill material with optimal mix proportion was studied by TGA, MIP, SEM–EDS and XRD. The results show that the mix proportion of CSW with the optimal comprehensive index is coal gangue (CG): coal fly ash (CFA): DG: FBS: GFS = 1:1.5:0.2:0.1:0.1, the mass concentration is 78%, and ordinary Portland cement (OPC)/CSW = 7.5%. The weight loss phenomenon of the backfill material with the optimal mix proportion occurs continuously during the heating process, mainly due to the evaporation of crystal water, structural water and hydroxyl water. There are dense narrow-necked pores in the backfill material, and the pore connectivity is poor. There is no hydration reaction occurs between CSW particles, and the strength increase of the backfill material mainly depends on the hydration reaction of cement. In ettringite, part of Al_2_O_3_ is replaced by SiO_2_, and part of CaSO_4_ is replaced by CaCO_3_. This study provides a reference for the engineering application of underground backfilling with multi-source CSW.

## Introduction

Ningdong Energy and Chemical Base (Ningdong Base) is located in the Energy Golden Triangle of the Yellow River Basin in western China, making important contributions to China's energy security^1,2^. However, a large amount of multi-source CSW mainly composed of CG, CFA, DG, GFS and FBS^3,4^ has an adverse impact on the local ecological environment^5,6^. Local companies urgently need to find green transformation methods for large-scale disposal of multi-source CSW. The underground backfilling is a green method for large-scale disposal of multi-source CSW, which can not only effectively reduce surface subsidence, but also significantly reduce the accumulation of solid waste on the surface and its pollution to the ecological environment^7,8^.

In the past, common backfill materials often consisted of one or two types of solid waste, such as CG, CFA, tailings, etc^9–11^, but there are few application cases of multi-source CSW backfill materials. In large energy and chemical bases in China, there are many types of CSW and the amounts are uneven^2^. Large-scale, effective and low-cost are a practical problem faced by coal mines for backfilling multi-source CSW. Therefore, it is first necessary to solve the material mix proportion of multi-source CSW for underground backfilling.

For composite backfill materials composed of various solid wastes, the mix proportion and performance profoundly affect the backfill efficiency, effect and benefit^12–14^. Many scholars have used different methods to study the mix proportion and performance of different backfill materials. Djurdevac et al.^15^ studied the effect of material ratio on the strength of backfill body based on the experimental method; Feng et al.^16^ studied the ratio and properties of gangue-waste concrete cemented paste backfill materials by response surface method; Ai et al.^17^ study the effects of mass fraction and sand cement ratio on the properties of paste backfill materials by the uniform design method; Wu et al.^18^ optimized the proportion of cemented backfill materials for tailings based on response surface and satisfaction function methods; Zhang et al.^19^ predicted and optimized the ratio and performance of tailings backfill materials based on the BP neural network model; Wen et al.^20^ used the multi-objective comprehensive evaluation method to develop the composite cementitious material and optimize the backfill slurry ratio; Li et al.^21^ used the game tree analysis method to solve the high-stage backfill body proportion optimization problem and applied it in practice. Aneke et al.^22,23^ studied the performance and microstructure of fly ash as a roadbed backfill material, providing reference for the study of the performance of mining solid waste backfill materials. Other scholars have used different methods to optimize the proportioning and performance of backfill materials with different types of solid waste^24–26^.

Although there also have been research results on the multi-source CSW for underground backfilling in recent years, for example, Zhang et al.^27,28^ used orthogonal experiments to study the mix proportion and mechanical properties of multi-source coal-based solid waste backfill materials, Wei et al.^29^ used RSM to study the mix proportions of four types of coal-based solid waste, Zhao et al.^30,31^ researched on the effects and proportions of four types of multi-source CSW, the current research and application are still not extensive and in-depth enough. Moreover, five kinds of CSW are used to prepare underground backfilling materials, with many types, complex compositions and unknown properties. Without mix proportion research, there will be great blindness in the on-site application of multi-source CSW. Using a single method to conduct mix proportion experiments is susceptible to the influence of multiple factors, and it is difficult to accurately and reliably obtain a material mix proportion with relatively optimal comprehensive indicators. Therefore, for multi-source CSW backfill materials, this article attempts to use two different methods to determine the mix proportion with optimal comprehensive indicators. Specifically, the RSM was used to determine the mix proportion of three kinds of CSW, and then the MDM was used to optimize the mix proportion of cementitious materials. Finally, the microstructure of the backfill material with the optimum mix proportion was analyzed. The research provides a reference for the engineering application of underground backfilling with multi-source CSW.

## Experimental materials

**Figure 1:**
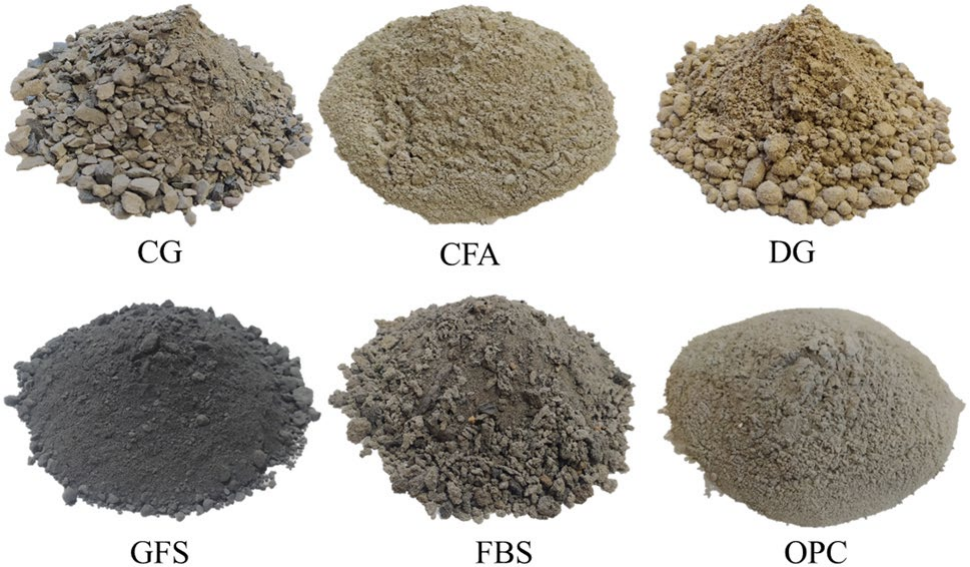
Experimental materials.

The backfill materials in this experiment are composed of CG, CFA, DG, GFS and FBS, and ordinary Portland cement (OPC) is used as cementing material. The experimental materials are shown in Fig. 1. It should be noted that the CSW in this experiment meets the requirements of the Chinese standard GB 18599-2020.

### Physical characteristics

#### CG

CG was taken from a coal mine in Ningdong Base, mainly composed of sandstone and shale, hard in quality and gray-black in appearance. The particle size of the original gangue is different. The maximum particle size of CG exceeds 100 mm, and the minimum particle size of CG is less than 5 mm. The crushed gangue was sieved, and the CG with a particle size of less than 15 mm was as the experimental material. The particle size distribution is shown in Table 1, the mass proportion of CG with a particle size range of 0–2.5 mm is the largest, which is 45.86%, and that of CG with a particle size range of 2.5–5 mm is the smallest, which is 13.38%.

**Table 1:** Particle size distribution of CG.

Particle size range	0–2.5/mm	2.5–5/mm	5–8/mm	8–15/mm
Mass proportion	45.86%	13.38%	26.34%	14.12%

#### CFA

**Figure 2:**
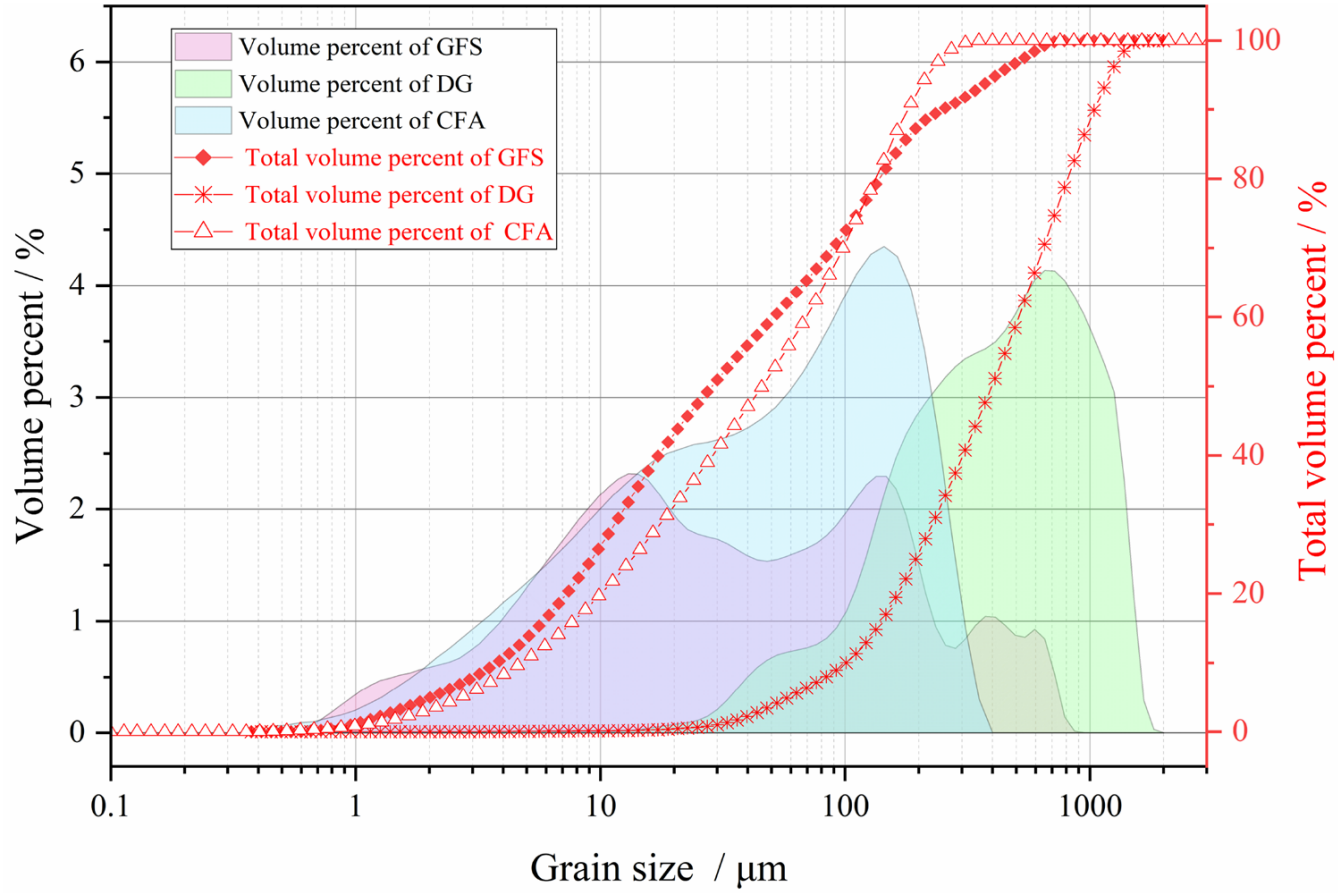
Particle size distribution of CFA, DG and GFS.

The CFA is the secondary fly ash discharged from a power plant in Ningdong Base. It is grayish-white in appearance, the fineness is about 20, loss on ignition is 2.5%, the specific surface area is 481.2 m^2^/kg, and moisture content is less than 1%, powdery, and has a certain water absorption. As shown in Fig. 2, the particle size range of CFA is basically between 0.1 and 300 μm, and the total volume percent with a particle size of less than 200 μm is about 95%.

#### DG

The DG is taken from a power plant in Ningdong Base. Its appearance is dark yellow, the moisture content is about 10%, massive and wet powder. As can be seen from Fig. 2, the result shows that the particle size range of DG is 20-2000 μm. The volume percent with a particle size of about 700 μm accounts for the largest proportion, more than 4.1%, the total volume percent with a particle size of less than 700 μm is about 70%.

#### GFS

The GFS is taken from a coal chemical plant in Ningdong Base. It is black in appearance, with a large carbon content, dry, mainly lumpy, and can be well dissolved with water. The particle size distribution curve of GFS showed a multimodal shape, the particle size range of DG is 0.4-1000 μm. The volume percent with a particle size of about 15 μm and 150 μm accounts for the largest proportion, more than 2.4% and 2.3% respectively.

#### FBS

The FBS is taken from a power plant in Ningdong Base. Its appearance is gray and brownish gray, lumpy and granular. The maximum particle size is more than 50 mm and the minimum particle size is less than 1 mm. The surface of the lumpy FBS is uneven, with many holes and pits. FBS with a particle size of less than 2.5 mm is as the experimental material.

#### OPC

The OPC produced by a cement plant in Huainan was used in the experiment, and the type of OPC is P.O32.5. It is gray-white in appearance, powdery, the fineness is about 16, and the volume with a particle size of less than 60 μm accounts for more than 85% of the total volume.

### Mineral composition

**Figure 3:**
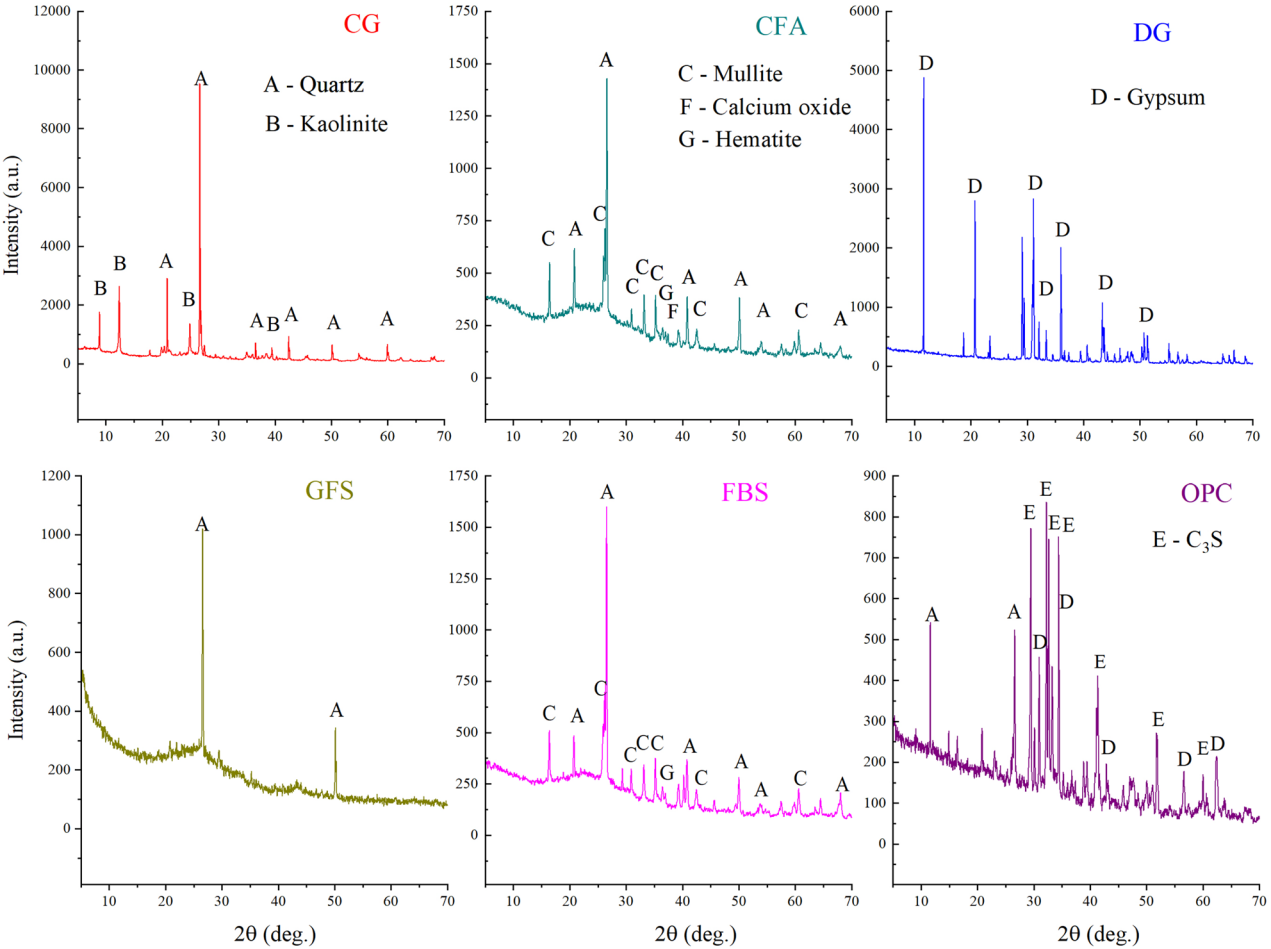
XRD results of experimental materials.

The XRD test results of the experimental materials are shown in Fig. 3. The main mineral components of CG are quartz (SiO_2_) and kaolinite (Al_2_Si_2_O_5_(OH)_4_). The main mineral components of CFA are quartz and mullite(Al_2_SiO_5_), and a small amount of calcium oxide (CaO) and hematite (Fe_2_O_3_). The main mineral component of DG is gypsum (CaSO_4_·2H_2_O). The main mineral component of GFS is quartz. The main mineral components of FBS are quartz and mullite, and a small amount of hematite. The main mineral component of OPC is C_3_S (Ca_3_SiO_5_), and a small amount of gypsum.

## Mix proportion experiment of solid waste

### RSM-BBD experimental scheme

There are many studies on CG, CFA and OPC as backfill materials^10,32,33^, so they are no longer taken as the research object in RSM experiment, instead of DG, FBS and GFS. The early strength (strength of 1 day, 3 days and 7 days) is as the response target, which is designed and analyzed by BBD in RSM. In the experimental scheme, the amount of CG was used as the benchmark, and the relative mass ratio was fixed as 1, and the materials are weighed according to the mass ratio of solid waste to CG. The mass ratios of DG, FBS and GFS to CG are DG/CG = 0.2, 0.3 and 0.4, FBS / CG = 0.1, 0.15 and 0.2, GFS/CG = 0.1, 0.2 and 0.3 respectively. By referring to relevant literature^34,35^ and considering the backfilling cost, it is determined that the mass ratio of CFA to CG is fixed as CFA/CG = 0.4, the mass ratio of OPC to CSW is fixed as OPC/CSW = 4%, and the mass concentration is fixed at 80%. The BBD mode was used for the RSM experimental design, and the design table is shown in Table 2.

**Table 2:** RSM-BBD experimental scheme.

No.	DG/CG	FBS/CG	GFS/CG
1	0.4	0.15	0.3
2	0.3	0.2	0.1
3	0.2	0.15	0.3
4	0.4	0.2	0.2
5	0.4	0.1	0.2
6	0.3	0.15	0.2
7	0.2	0.1	0.2
8	0.2	0.15	0.1
9	0.3	0.15	0.2
10	0.3	0.2	0.3
11	0.3	0.15	0.2
12	0.3	0.1	0.3
13	0.3	0.15	0.2
14	0.3	0.1	0.1
15	0.4	0.15	0.1
16	0.3	0.15	0.2
17	0.2	0.2	0.2

The water consumption of backfill materials in each experimental group is calculated by the mass concentration equation, as shown in Eq. (1).

1 $$P=\frac{S}{S+W}\times 100\%$$

where *P* is the mass concentration of the backfill material; *S* is the mass of solids in the backfill material, which is the amount of CSW and OPC; *W* is the water consumption of the backfill material.

### Experimental method

The preparation method and compressive strength test method of the backfill material refer to the Chinese standard "*Standard for Test Methods of Basic Performance of Building Mortar*" (JGJ/T70-2009). The curing conditions simulate the underground environment, the temperature is 25 ± 2 °C, and the relative humidity is 85 ± 5%. The curing age was 1 day, 3 days, and 7 days, respectively. Three specimens in each group were made at 1 day, 3 days and 7 days, and the average strength was as the compressive strength.

### Experimental results

#### Regression fit analysis

The experimental results are shown in Table 3. The data in the table are fitted and analyzed by multiple regression, and the strength response surface functions of 1 day, 3 days and 7 days are obtained.

**Table 3:** Early strength of backfill specimen.

No.	1d/MPa	3d/MPa	7d/MPa
1	0.25	0.39	0.61
2	0.29	0.39	0.66
3	0.26	0.39	0.57
4	0.26	0.35	0.62
5	0.24	0.34	0.48
6	0.3	0.4	0.62
7	0.37	0.5	0.78
8	0.33	0.49	0.72
9	0.26	0.41	0.64
10	0.24	0.35	0.52
11	0.29	0.38	0.65
12	0.23	0.36	0.63
13	0.29	0.43	0.64
14	0.31	0.41	0.59
15	0.3	0.42	0.55
16	0.28	0.4	0.63
17	0.24	0.42	0.56

As can be seen from Table 3 that the early strength of the backfill specimen increases with the increase of the curing age. The early strength of the specimens in Experiment No. 7 is the largest, while that of the specimens in Experiment No.5 is the lowest. The difference between the mix proportions of the two experimental groups is the DG content. It can be seen that the increase in DG is not conducive to the increase in early strength and this result is consistent with the results in a previously published article^29^. The early strength of the specimens did not exceed 1 MPa, because the cement content in the backfill materials was small and the content of hydration reaction products in the solid waste materials was low. This result has also been verified in published articles^27,28^. In this article, when the cement content is ≤ 5%, the early strength of multi-source CSW backfill materials is low, with the 3-day strength and 7-day strength ranging from 0.15–0.45 MPa to 0.27–0.97 MPa respectively, and GFS and DG are not conducive to increasing the strength of the backfill specimens. The negative effect of GFS on the strength of multi-source CSW backfill materials has also been verified in public articles^29^. This study pointed out that GFS is suspended in the backfill material, resulting in uneven distribution of slurry aggregates and weakening the strength, and the strength increase of multi-source CSW backfill materials is basically attributed to the hydration reaction of cement additives.

To display the functional relationship between the three types of CSW content and early strength, and to predict the early strength of the backfill material at different amounts of three types of CSW, the strength response surface function at different curing age is obtained through the nonlinear regression fitting:

1 day:

Y_1_ = 0.28–0.019X_a_–0.015X_b_–0.031X_c_ + 0.037 X_a_X_b_ + 5 × 10^−3^X_a_X_c_ + 7.5 × 10^−3^X_b_X_c_ + 5.5 × 10^−3^X_a_^2^–0.012X_b_^2^–4.5 × 10^−3^X_c_^2^ (R^2^ = 0.873).

3 days:

Y_3_ = 0.4–0.038X_a_–0.012X_b_–0.028X_c_ + 0.022X_a_X_b_ + 0.018X_a_X_c_ + 2.5 × 10^−3^X_b_X_c_ + 0.022X_a_^2^–0.023X_b_^2^–3.25 × 10^−3^X_c_^2^ (R^2^ = 0.841).

7 days:

Y_7_ = 0.64–0.046X_a_–0.015X_b_–0.024C + 0.09X_a_X_b_ + 0.053X_a_X_c_–0.045X_b_X_c_–6.75 × 10^−3^X_a_^2^–0.019X_b_^2^–0.017X_c_^2^ (R^2^ = 0.972).where X_a_, X_b_ and X_c_ are DG/CG, FBS/CG and GFS/CG, respectively.

Each function is a ternary quadratic equation. The correlation coefficient R^2^ in the 3-day strength function is the smallest, 0.841, and the correlation coefficients R^2^ in the other function are all greater than 0.87, indicating that the regression function of early strength has a good correlation with the experimental results, the fitting degree is high, and it has a good predictability for the early strength.

#### ANOVA

The strength at different curing ages is by Analysis of Variance (ANOVA), and the P value and F value in the ANOVA results are listed in Table 4.

**Table 4:** *P* and F value in ANOVA results.

Source	1d		3d		7d	
	*P*	F	*P*	F	*P*	*F*
Model	0.0190	5.34	0.0381	4.10	0.0001	27.08
Lack of fit	0.1793	2.72	0.1222	3.64	0.0925	4.42

*P* ≤ 0.01 means highly significant; *P* ≤ 0.05 means significant; *P* > 0.05 means not significant.

It can be seen from Table 4 that the F values in the 1-day, 3-day, and 7-day strength regression models are 5.34, 4.1, and 27.08, respectively, and the values are all greater than F_0.95_ (3, 13) = 3.42, indicating that the model is highly significant. The P values of the strength regression models at different curing ages were 0.019, 0.0381, and 0.0001, all of which were less than 0.05, indicating that the regression effects of each model were significant, with high reliability and statistical significance. The P values of the lack of fit are 0.1793, 0.1222, and 0.0925, which are greater than 0.05, and the F values are 2.72, 3.64 and 4.42 respectively, which are less than F_0.05_(3, 4) = 6.59. The lack of fit is not significant, indicating that the model is appropriate.

#### Optimal mix proportion of solid waste

The mix proportion of DG, FBS and GFS was optimized by the regression model, and the result was DG/CG = 0.2, FBS/CG = 0.1 and GFS/CG = 0.1, respectively. It is predicted that the strength values of the backfill with this optimal mass ratio at 1 day, 3 days and 7 days are 0.39 MPa, 0.52 MPa and 0.78 MPa respectively. To verify the reliability of the regression model prediction, the backfill specimen was made with the mix proportion of CG: CFA: DG: FBS: GFS = 1:0.4:0.2:0.1:0.1, and the average compressive strength of the specimen with curing age of 1 day, 3 days and 7 days was tested. The results show that the average strength values of the backfill specimens with the optimal mix proportion of CSW at 1 day, 3 days and 7 days are 0.38 MPa, 0.51 MPa and 0.81 MPa, respectively, which are less than 4% different from the prediction values at each age in the strength regression model. It can be seen that the strength regression model has guiding significance for the optimization and prediction of CSW mix proportion under the same conditions. Therefore, DG/CG = 0.2, FBS/CG = 0.1 and GFS/CG = 0.1 are the optimal mix proportion of the three CSW materials in the RSM experiment.

## Mix proportion of cementitious materials

The optimal mix proportion of DG, FBS and GFS is determined by RSM- BBD experiment, but the strength of CSW materials with the above mix proportion is low, which can not meet the mine's requirements for the strength of the backfill. In this experiment, based on fixing the proportion of DG, FBS and GFS, the early strength of CSW backfill material was improved by changing the content of cementitious material. In the process of underground mining, the pressure period of the working face is generally more than 4–5 days, and some even more than 7–10 days. Therefore, in order to support the roof timely and effectively, the strength of the backfill at 3 days and 7 days was taken as the target optimization object in this experiment^36^.

### Experimental scheme

CFA and OPC, as a cementing material with a better cementation effect, are of great help in improving the mechanical properties^37,38^. In this experiment, the mass ratio of CFA to CG was set as CFA/CG = 0.5, 0.75, 1.0, 1.25 and 1.5, corresponding to the proportion of CFA in CSW material were CFA/CSW = 26.23%, 34.88%, 41.67%, 47.17% and 51.72% respectively. The mass ratio of OPC to CSW was set as OPC/CSW = 5%, 7.5%, 10% and 12.5%. The mass concentration was fixed at 78%. The specific experimental scheme is shown in Table 5.

**Table 5:** Mix proportion of cementitious materials.

No.	CG	CFA/CG (CFA/CSW)	DG/CG	FBS/CG	GFS/CG	OPC/CSW/%
1	1	0.5 (26.23%)	0.2	0.1	0.1	5
2	1	0.75 (34.88%)	0.2	0.1	0.1	5
2	1	1.0 (41.67%)	0.2	0.1	0.1	5
4	1	1.25 (47.17%)	0.2	0.1	0.1	5
5	1	1.5 (51.72%)	0.2	0.1	0.1	5
6	1	0.5 (26.23%)	0.2	0.1	0.1	7.5
7	1	0.75 (34.88%)	0.2	0.1	0.1	7.5
8	1	1.0 (41.67%)	0.2	0.1	0.1	7.5
9	1	1.25 (47.17%)	0.2	0.1	0.1	7.5
10	1	1.5 (51.72%)	0.2	0.1	0.1	7.5
11	1	0.5 (26.23%)	0.2	0.1	0.1	10
12	1	0.75 (34.88%)	0.2	0.1	0.1	10
13	1	1.0 (41.67%)	0.2	0.1	0.1	10
14	1	1.25 (47.17%)	0.2	0.1	0.1	10
15	1	1.5 (51.72%)	0.2	0.1	0.1	10
16	1	0.5 (26.23%)	0.2	0.1	0.1	12.5
17	1	0.75 (34.88%)	0.2	0.1	0.1	12.5
18	1	1.0 (41.67%)	0.2	0.1	0.1	12.5
19	1	1.25 (47.17%)	0.2	0.1	0.1	12.5
20	1	1.5 (51.72%)	0.2	0.1	0.1	12.5

### Experimental method

**Figure 4:**
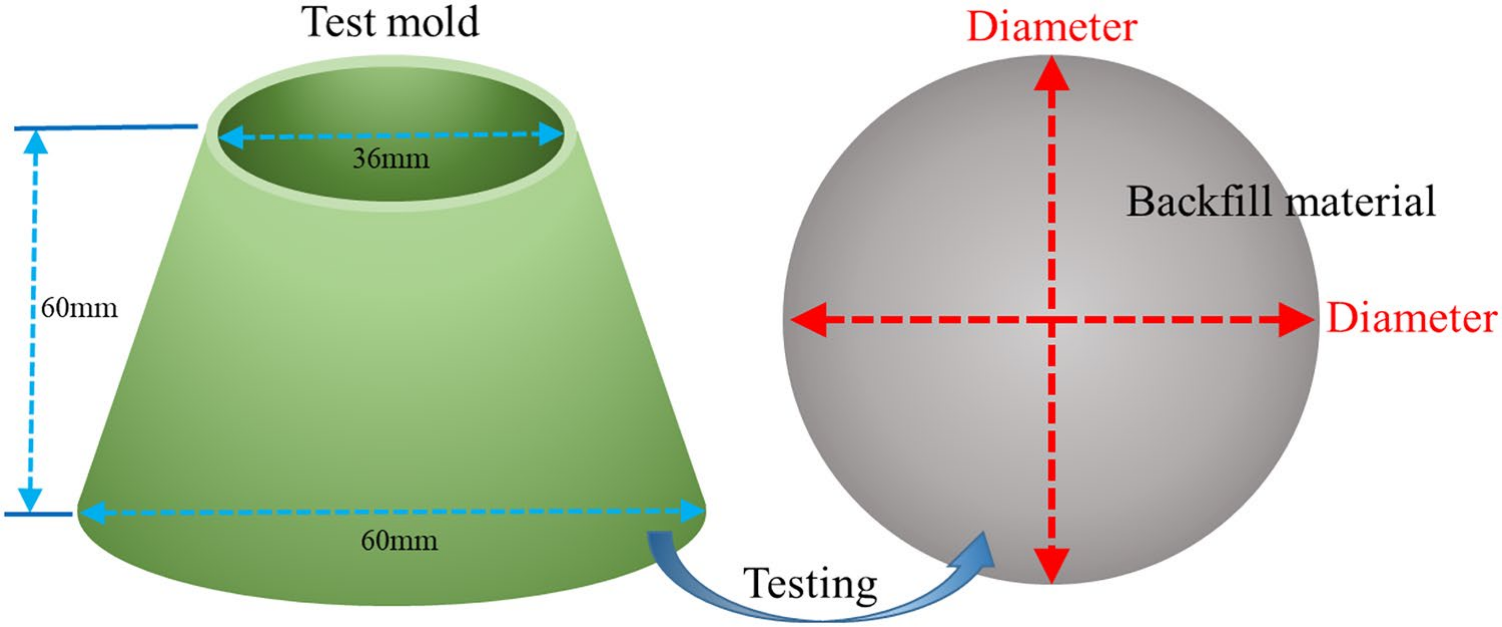
Backfill material fluidity test.

The preparation and the strength test method of the backfill specimen refer to the above RSM-BBD experiment. In this experiment, the fluidity and bleeding rate are tested. The fluidity of the backfill material is the average diameter of the material flowing freely on the glass plane^26^. The test method refers to the Chinese standard "*Test Method for the Homogeneity of Concrete Admixtures*" (GBT8077-2000), as shown in Fig. 4.

The bleeding rate (also called decantation rate) is the ratio of the bleeding volume in the static slurry to the initial volume of the slurry. Its test method refers to the literature^39^ and the Chinese standard "*Prestressed Pore Grouting Agent*" (GB /T 25,182–2010). The calculation equation of bleeding rate is as follows:

2 $$b=\frac{{V}_{1}}{{V}_{0}}\times 100\%$$

where *b* is the bleeding rate of the static backfill material; *V*_1_ is the bleeding volume of the backfill material; *V*_0_ is the initial volume of the backfill material.

### Analysis of experimental results

#### Fluidity and bleeding rate

**Figure 5:**
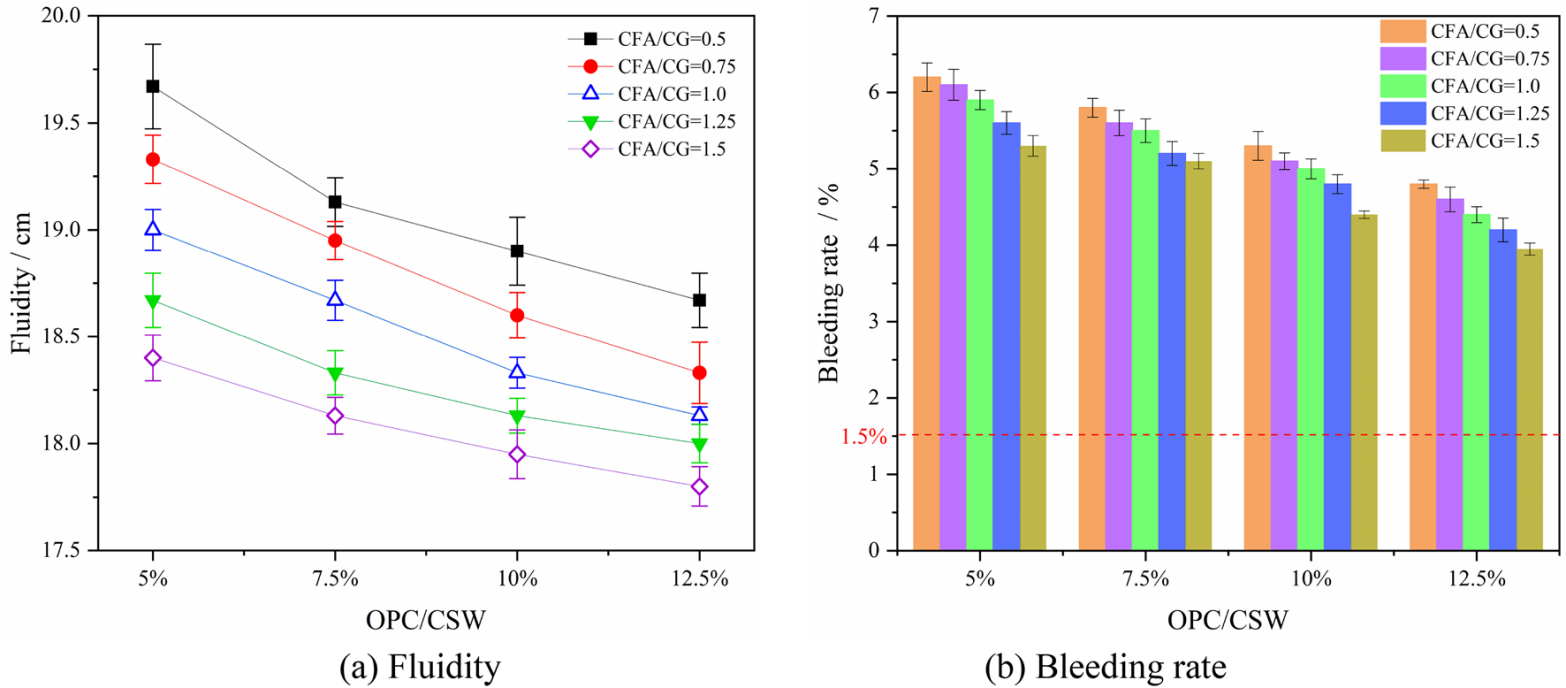
Fluidity and bleeding rate of backfill material.

The fluidity and bleeding rate of backfill materials in different experimental groups were tested, and the results are shown in Fig. 5.

As can be seen from Fig. 5a that with the increase of OPC content, the fluidity of backfill materials shows a slow downward trend. When OPC/CSW increased from 5 to 12.5%, the fluidity of backfill materials with CFA/CG of 0.5, 1.0 and 1.5 decreased from 19.67 cm, 19 cm and 18.4 cm to 18.67 cm, 18.13 cm and 17.8 cm respectively, with a decrease of 5.08%, 4.58% and 3.26% respectively. The effect of CFA on the fluidity of the backfill material is the same as that of OPC, and the increase of its content has a negative effect on the fluidity. When CFA / CSW increased from 26.32% (CFA/CG = 0.5) to 41.67% (CFA/CG = 1.5), the fluidity of backfill materials with OPC / CSW of 5%, 7.5%, 10% and 12.5% decreased by 6.46%, 5.23%, 5.03% and 4.66% respectively.

The influence of the content of OPC and CFA on the bleeding rate of backfill materials also shows an obvious negative effect. When OPC / CSW increased from 5 to 12.5%, the bleeding rate of backfill materials with CFA / CG of 0.5, 1.0 and 1.5 decreased from 6.2%, 5.9% and 5.3% to 4.8%, 4.4% and 3.95% respectively, and the maximum bleeding rate decreased by more than 1.5%; When CFA / CSW increased from 26.32% (CFA/CG = 0.5) to 51.72% (CFA / CG = 1.5), the fluidity of backfill materials with OPC/CSW of 5%, 7.5%, 10% and 12.5% decreased from 6.2%, 5.8%, 5.3% and 4.8% to 5.3%, 5.1%, 4.4% and 3.95% respectively.

#### Early strength

**Figure 6:**
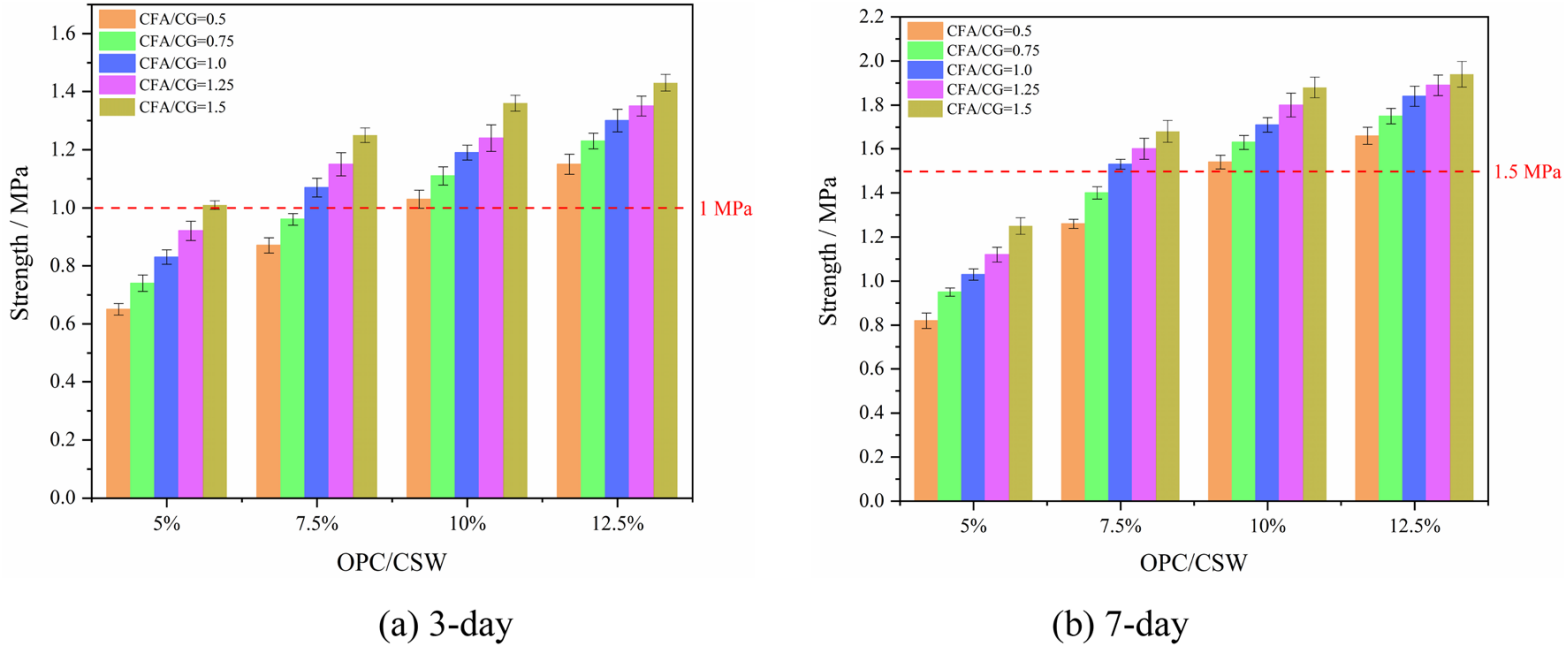
Early strength of backfill specimens.

The early strength of the specimens was obtained by uniaxial compression test, as shown in Fig. 6.

As can be seen from Fig. 6 that with the increase of OPC content, the 3-day and 7-day strength of the backfill specimen increases significantly. When OPC/CSW increased from 5 to 12.5%, the 3-day strength of backfill specimens with CFA/CG of 0.5, 1.0 and 1.5 increased from 0.65 MPa, 0.83 MPa and 1.01 MPa to 1.15 MPa, 1.3 MPa and 1.43 MPa. The 7-day strength increased from 0.85 MPa, 1.03 MPa and 1.25 MPa to 0.85 MPa, 1.03 MPa and 1.25 MPa. The increase of CFA content is also beneficial to the growth of early strength of the backfill. When the CFA/CSW increased from 26.32% (CFA/CG = 0.5) to 51.72% (CFA/CG = 1.5), the 3-day strength and 7-day strength of the backfill specimens increase by more than 24.35% and 16.87% respectively. The 3-day and 7-day strength increases with the increase of CFA content.

## Comprehensive evaluation of mix proportion

### MDM

The mix proportion optimization of backfill materials involves not only its fluidity, bleeding rate and early strength, but also its preparation cost. It is a multi-objective decision-making model, which is suitable for the comprehensive evaluation and optimization model. In this regard, *m* indicators are used to comprehensively evaluate *n* schemes, and a target eigenvalue matrix is established^30,40^:

$${\varvec{X}}=\left[\begin{array}{cccc}{x}_{11}& {x}_{12}& \cdots & {x}_{1n}\\ {x}_{21}& {x}_{22}& \cdots & {x}_{2n}\\ \vdots & \vdots & \vdots & \\ {x}_{m1}& {x}_{m2}& \cdots & {x}_{mn}\end{array}\right]={\left({x}_{ij}\right)}_{m\times n}$$

According to the types of different indicators, the target eigenvalue matrix is normalized by Eq. (3)-(5), and the target relative superiority matrix *R* is as follows:

$${\varvec{R}}=\left[\begin{array}{cccc}{r}_{11}& {r}_{12}& \cdots & {r}_{1n}\\ {r}_{21}& {r}_{22}& \cdots & {r}_{2n}\\ \vdots & \vdots & \vdots & \\ {r}_{m1}& {r}_{m2}& \cdots & {r}_{mn}\end{array}\right]={\left({r}_{ij}\right)}_{m\times n}$$

There are three main types of target relative superiority, namely, the bigger the better type, the moderately intermediate type, and the smaller the better type, and each type is calculated by its equation.

The larger the better type:

3 $${r}_{ij}=\frac{{x}_{ij}-min\left({x}_{ij}\right)}{max\left({x}_{ij}\right)-min\left({x}_{ij}\right)}$$

The moderate intermediate type:

4 $${r}_{ij}=1-\left|\frac{{x}_{ij}-{y}_{i}}{max\left({x}_{ij}\right)-min\left({x}_{ij}\right)}\right|$$

The smaller the better type:

5 $${r}_{ij}=\frac{max\left({x}_{ij}\right)-{x}_{ij}}{max\left({x}_{ij}\right)-min\left({x}_{ij}\right)}$$

where, *r*_*ij*_ represents the target eigenvalue after normalization of the *i*-th index in Scheme *j*; *y*_*i*_ is the ideal value of the *i-*th index; *i* = 1, 2, …, *m*; *j* = 1, 2, …, *n*.

The weight is determined according to the importance of the evaluation index, and the weight matrix is established as follows:

$$w=\left[\begin{array}{cccc}{w}_{1}& {w}_{2}& \cdots & {w}_{i}\end{array}\right]$$

where *w*_*i*_ represents the weight of the *i*-th evaluation index.

According to the principle that the square sum of weighted distance superior Euclidean distance and weighted distance inferior Euclidean distance is the smallest, the relative superiority is obtained, as shown in Eq. (6). Finally, the scheme with a relatively optimal comprehensive index is selected by comparing the relative superiority of each scheme.

6 $${u}_{j}=1/\left\{1+\frac{\sum_{i=1}^{m} {\left[{w}_{i}\left({a}_{i}-{r}_{ij}\right)\right]}^{2}}{\sum_{i=1}^{m} {\left[{w}_{i}\left({r}_{ij}-{b}_{i}\right)\right]}^{2}}\right\}=1/\left\{1+\frac{\sum_{i=1}^{m} {\left[{w}_{i}\left(1-{r}_{ij}\right)\right]}^{2}}{\sum_{i=1}^{m} {\left({w}_{i}{r}_{ij}\right)}^{2}}\right\}$$

### Mix proportion optimization

The backfill material can be transported into the ground smoothly and has a good supporting effect, which needs to meet certain indicators. According to references^20,24,41,42^, the CSW backfill material is pumped into the underground space in the form of fluid or paste, and the reasonable range of its bleeding rate is 1.5–20%; The mine’s requirements for the early strength of the backfill are that the 3-day strength *R*_3d_ ≥ 1 MPa, and the 7-day strength *R*_7d_ ≥ 1.5 MPa. According to Fig. 5a,b, the bleeding rate of the backfill materials in each scheme is greater than 1.5%, which is within the reasonable range of the bleeding rate. However, the 3-day and 7-day strengths of Experiment No.1–No.7 are lower than the 1 MPa and 1.5 MPa, respectively, which do not meet the mine's requirements for the early strength of the backfill. Therefore, there are thirteen groups of experiments to meet the requirements for fluidity and early strength of mine backfill material. The fluidity, bleeding rate, 3-day strength, 7-day strength and preparation cost are taken as the evaluation indexes to comprehensively evaluate and optimize the 8 groups of experiments that meet the requirements, the target eigenvalues are shown in Table 6. Among them, the preparation cost of backfill material is calculated according to the price of each material required to prepare backfill materials per unit mass, ignoring the transportation cost of each material, which is mainly composed of the cost of OPC and water.

**Table 6:** Target eigenvalues of the thirteen groups of experiments.

No.	Fluidity/cm	Bleeding rate/%	*R*_3d_/MPa	*R*_7d_/MPa	Cost/(RMB/t)
8	18.67	5.5	1.07	1.53	52
9	18.33	5.2	1.15	1.6	55
10	18.13	5.1	1.25	1.68	57
11	18.9	5.3	1.03	1.54	54
12	18.6	5.1	1.11	1.63	58
13	18.33	5	1.19	1.71	62
14	18.13	4.8	1.24	1.8	65
15	17.95	4.4	1.36	1.88	67
16	18.67	4.8	1.15	1.66	64
17	18.33	4.6	1.23	1.75	68
18	18.13	4.4	1.3	1.84	72
19	18	4.2	1.35	1.89	75
20	17.8	3.95	1.43	1.94	77

The target eigenvalue matrix *X* of thirteen groups of experiments is as follows:

$${X}_{5\times 8}=\left[\begin{array}{ccccccccccccc}18.67& 18.33& 18.13& 18.9& 18.6& 18.33& 18.13& 17.95& 18.67& 18.33& 18.13& 18& 17.8\\ 5.5& 5.2& 5.1& 5.3& 5.1& 5& 4.8& 4.4& 4.8& 4.6& 4.4& 4.2& 3.95\\ 1.07& 1.15& 1.25& 1.03& 1.11& 1.19& 1.24& 1.36& 1.15& 1.23& 1.3& 1.35& 1.43\\ 1.53& 1.6& 1.68& 1.54& 1.63& 1.71& 1.8& 1.88& 1.66& 1.75& 1.84& 1.89& 1.94\\ 52& 55& 57& 54& 58& 62& 65& 67& 64& 68& 72& 75& 77\end{array}\right]$$

According to the types of evaluation indicators, the fluidity, 3-day strength and 7-day strength belong to the larger the better type; the bleeding rate within a reasonable range of 1.5%-20% can be regarded as the smaller the better type; the preparation cost belongs to the smaller the better type. Thus, the target eigenvalues are normalized by Eqs. (3) and (5), and the target relative superiority matrix *R* is as follows:

$${R}_{5\times 8}=\left[\begin{array}{ccccccccccccc}0.791& 0.482& 0.3& 1& 0.727& 0.482& 0.3& 0.136& 0.791& 0.482& 0.3& 0.182& 0\\ 0& 0.194& 0.258& 0.129& 0.258& 0.323& 0.452& 0.71& 0.452& 0.581& 0.71& 0.839& 1\\ 0.1& 0.3& 0.55& 0& 0.2& 0.4& 0.525& 0.825& 0.3& 0.5& 0.675& 0.8& 1\\ 0& 0.171& 0.366& 0.024& 0.244& 0.439& 0.659& 0.854& 0.317& 0.537& 0.756& 0.878& 1\\ 1& 0.88& 0.8& 0.92& 0.76& 0.6& 0.48& 0.4& 0.52& 0.36& 0.2& 0.08& 0\end{array}\right]$$

The preparation cost of backfill material is the most important in engineering practice, followed by 3-day and 7-day strength. Therefore, according to the importance of each evaluation index in engineering practice, the weights of fluidity* w*_1_, bleeding rate *w*_2_, 3-day strength *w*_3_, 7-day strength *w*_4_ and preparation cost *w*_5_ are determined to be 0.1, 0.05, 0.25, 0.15 and 0.5 respectively. The weight vectors of evaluation indexes are as follows:

$$w=\left[\begin{array}{ccccc}0.1& 0.05& 0.2& 0.15& 0.5\end{array}\right]$$

The relative superiority of 8 groups of experiments is calculated by Eq. (6), and the result is as follows:

$${u}_{j}=\left[\begin{array}{ccccccccccccc}0.816& 0.823& 0.841& 0.774& 0.735& 0.613& 0.485& 0.461& 0.474& 0.302& 0.202& 0.175& 0.2\end{array}\right]$$

Comparing the relative superiority of each experiment, it can be found that the relative superiority of Experiment No.10 is the largest, which is 0.841, which can be regarded as the optimal scheme obtained by MDM in the experiment. The mix proportion of cementitious material is CFA/CG = 0.5, OPC/CSW = 7.5%, the mix proportion of CSW backfill material is CG: CFA: DG: FBS: GFS = 1:1.5:0.2:0.1:0.1, and the mass concentration is 78%. In order to verify the reliability of the evaluation results, the CSW backfill material is prepared with the mix proportion of the optimal scheme, and the fluidity, bleeding rate and early strength are tested. The results showed that the fluidity of the backfill material was 18.05 cm, the bleeding rate was 5%, and the 3-day and 7-day strengths were 1.21 MPa and 1.63 MPa respectively, which is less than 5% different from the experimental results of the Experiment No.10 The experimental results are relatively reliable. The fluidity and early strength of the backfill material with the optimal mix proportion meet the theoretical requirements of the mine, and the comprehensive evaluation index is relatively optimal. The research results provide a theoretical reference for the engineering application of backfill materials.

## Microstructure

To further study the microstructure of the CSW backfill material with the optimal mix proportion, the thermal stability, pore structure, microscopic morphology and composition were tested and analyzed by Thermogravimetric Analysis (TGA), Mercury Intrusion Porosimetry (MIP), Scanning Electron Microscope- Energy Dispersive Spectromete (SEM–EDS) and X-ray diffraction (XRD).

### Testing method

(1) The reaction atmosphere used in the TGA test was nitrogen, the heating rate is 10 °C/min, the temperature range is 28–800 °C, the temperature resolution is 0.01 °C, the thermal resolution is 0.01 μV, and the mass sensitivity is 0.1 μg. (2) The AutoPore IV automatic mercury porosimeter was used in the MIP test, with a test aperture range of 0.003–1100 μm and high pressure of up to 33,000 psi. Based on the Washburn formula, the mercury volume in the intrusion sample pores under different pressures is measured, and the relationship curve between the mercury pressure and the intrusion mercury volume is obtained, and then the relevant pore data is obtained by the curve analysis. (3) In the SEM test, the acceleration voltage is 5 kV, the magnification is 20 k times, the scale is 2 μm, and the EDS point scan is used. (4) In the XRD test, the scanning speed is 4°/min, the range is 5–90°, the Cu target, the Ka radiation, and the continuous scanning method is adopted.

### Testing result

#### Thermal stability

**Figure 7:**
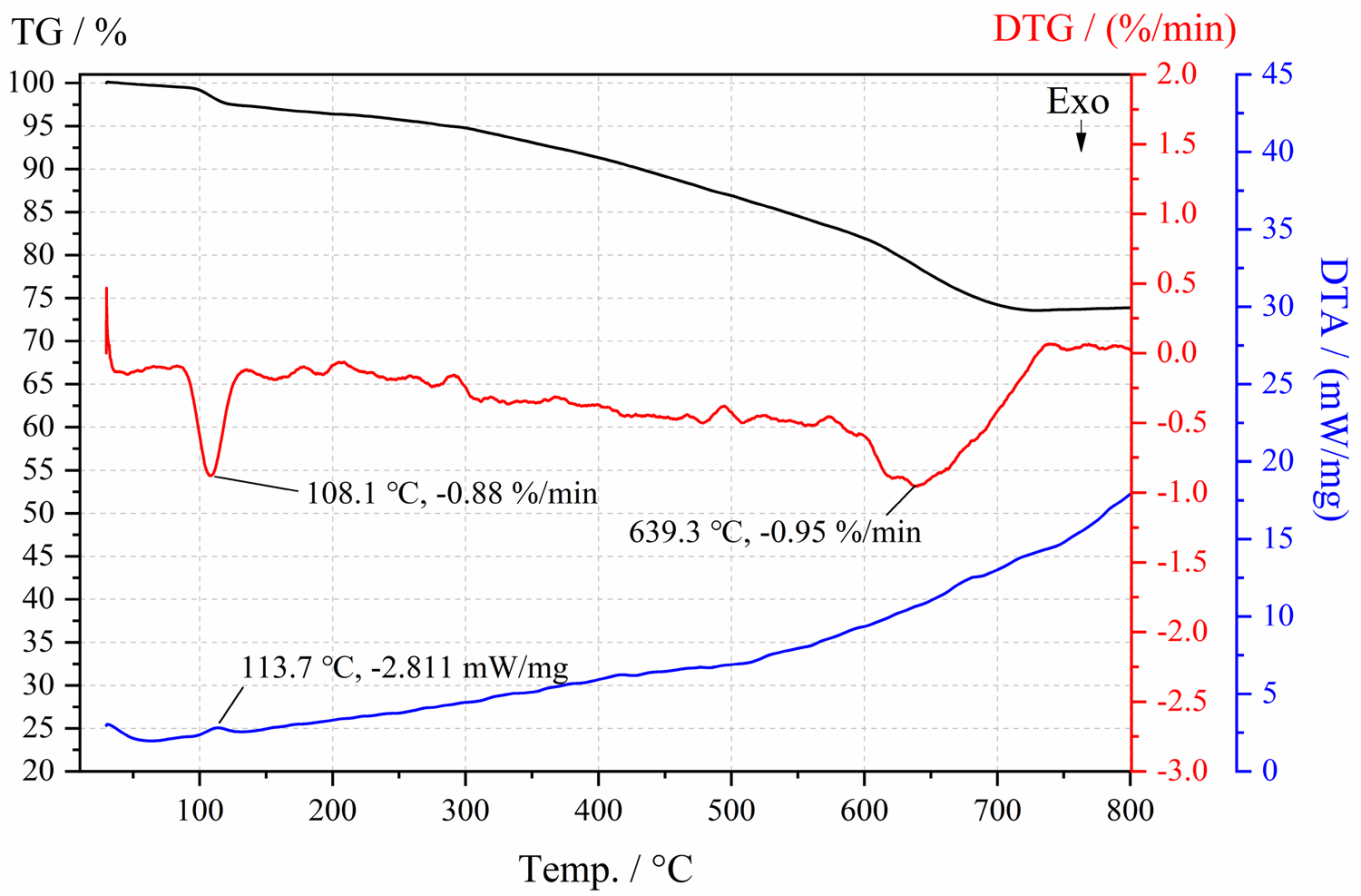
TGA test results.

The backfill specimen with the optimal mix proportion is tested by TGA, and the curves of TG, DTG and DTA are shown in Fig. 7.

As can be seen from Fig. 7, at 95–120 ℃, the TG curve decreased sharply, the DTG and DTA curves showed characteristic peaks, and the peak temperatures are 108.1 °C and 113.7 °C respectively, indicating that the evaporation of free water and the removal of crystal water occurred in the backfill specimen. The reaction process absorbed heat, and the loss rate of weight loss was fast, the loss rate reached the maximum at 108.1 ℃, which was -0.88% /min. When the temperature is higher than 120 ℃, the TG curve continues to decline, the DTG curve shows a fluctuating downward trend below 0%/min, and the DTA curve shows a slowly rising arc-like shape, indicating that with the increase of temperature, the weight loss rate in the backfill material gradually accelerates, and the process is generally exothermic. At 600–700 ℃, the TG curve shows an obvious sudden drop again, with a weight loss of about 6.5%. The DTG curve has a characteristic peak with a large width, and the peak value appears at 639.3 ℃, while the DTA curve has no obvious change. It is speculated that during this heating process, the products in the backfill material appear decomposition reaction and the removal of structural water and hydroxyl water in some substances, resulting in rapid weight loss of the material, with a maximum rate of − 0.95%/min. At 700–800 °C, the changes of TG and DTG curves tend to be stable, the weight loss of backfill materials tends to be less, and the loss rate is slow. At 800 ℃, the weight of the backfill material is 74% of the initial weight, and the total weight loss is about 26%.

#### Pore structure

**Figure 8:**
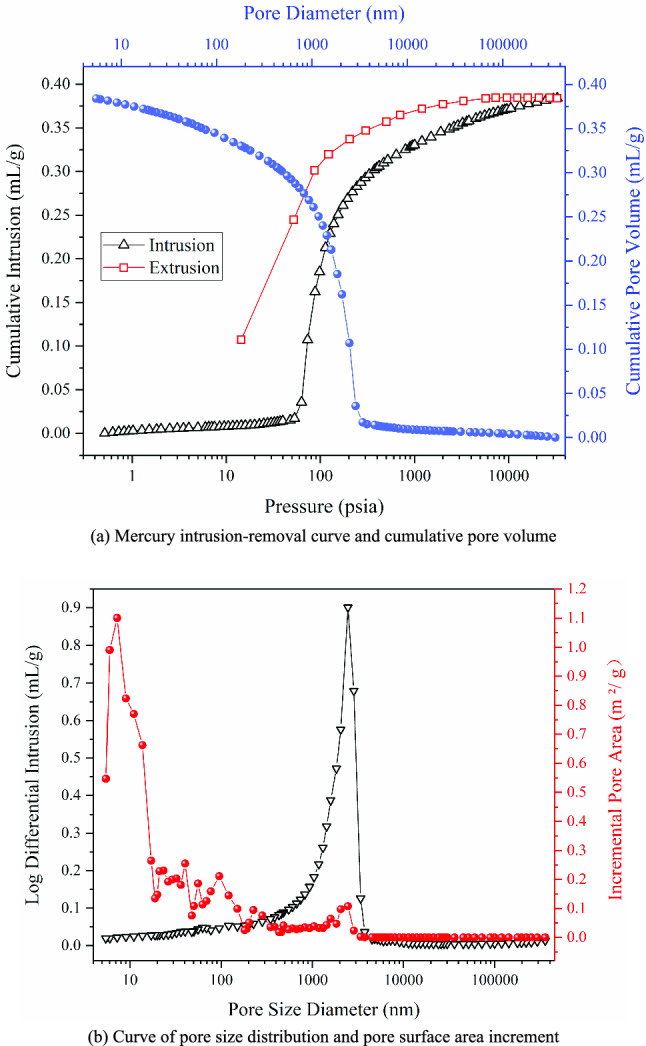
MIP test results.

The pore structure of backfill specimen with the optimal mix proportion is analyzed by MIP. The results are shown in Fig. 8.

As can be seen from Fig. 8a that the curve of mercury intrusion amount and pressure is similar to S-shaped: when the mercury intrusion pressure is less than 50 psia, the mercury intrusion amount changes gently; When the mercury intrusion pressure is between 50 and 300 psia, the mercury intrusion amount increases sharply, indicating that the pore volume content corresponding to the pressure greater than 50psia increases significantly; When the mercury intrusion pressure exceeds 300psia, the increase of the mercury intrusion amount becomes slow, the mercury enters into smaller pores. During the mercury removal process, the mercury removal curve and the mercury intrusion curve do not overlap, and the mercury removal curve is always above the mercury intrusion curve, indicating that there is mercury retention in the process, which is due to the dense narrow-necked pores in the specimen, with poor pore connectivity^43^. According to the cumulative pore volume curve, it can be seen that the pore diameter in the specimen is in the range of 4–4 × 10^5^ nm. When the pore diameter is less than 4000 nm, the decreasing trend of cumulative pore volume is like a parabola, and the cumulative pore volume decreased gradually when the pore diameter was greater than 4000 nm, indicating that the pore volume was mainly distributed in the pores with a pore diameter of less than 4000 nm.

It can be seen from Fig. 8b that the pore size distribution curve shows a single peak shape, and the change of pore volume in the range of 300–4000 nm is the most significant. indicating that there is a large distribution of pore diameter in this range, among which the pore volume with a pore diameter of about 2500 nm is the largest. According to the pore size division method of XOJIOT^44^, there are few micropores and transition pores in the backfill specimen, mainly medium and large pores. It can be seen from the pore surface area increment curve that the pore diameter has a peak at 7 nm, and the pore surface area increment with a pore diameter of less than 10 nm is obviously large, but the pore volume increase is small, indicating that the micro pores contribute the most to the pore surface area.

#### Micromorphology and composition

**Figure 9:**
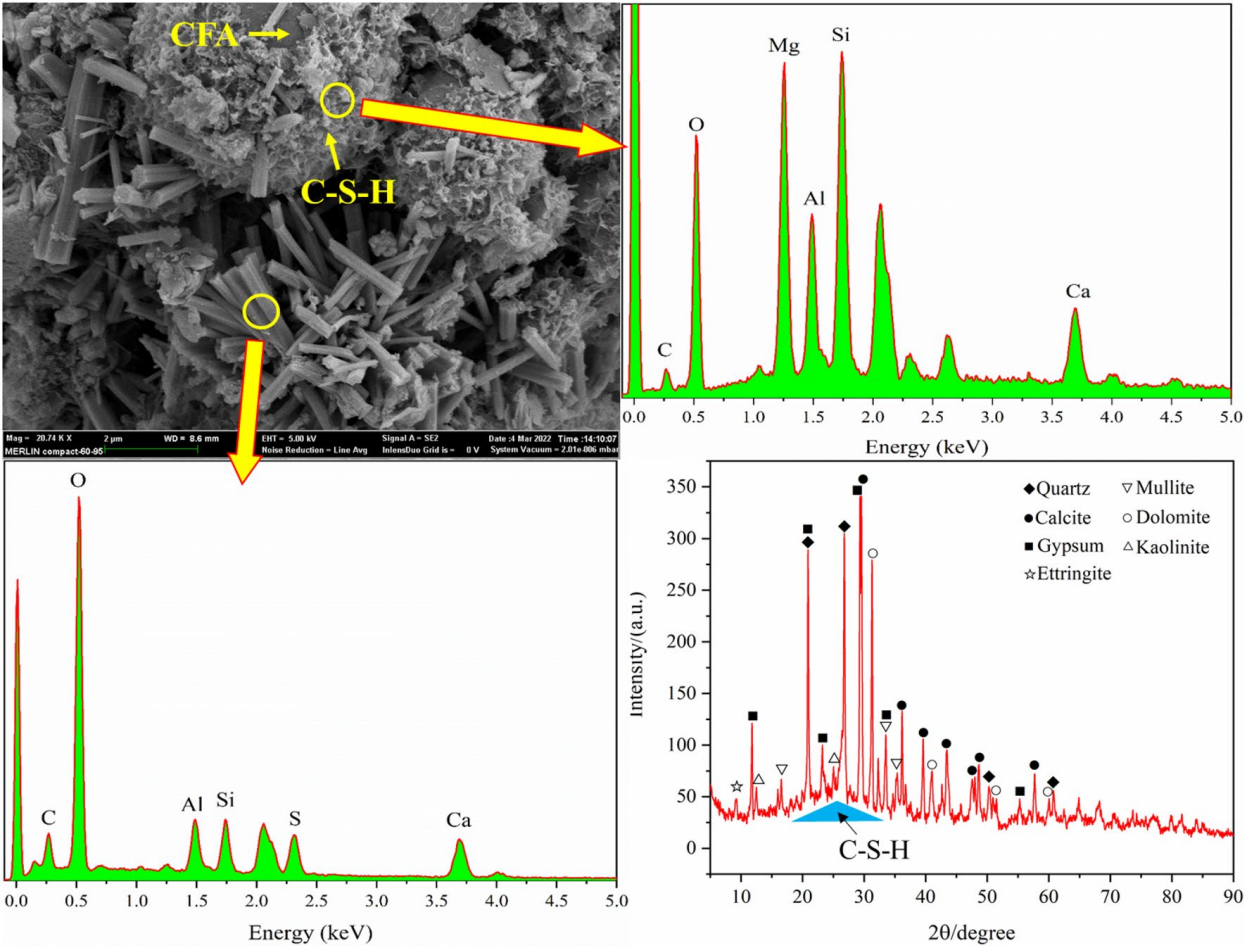
SEM–EDS and XRD test results.

The mineral composition and the micromorphology of the backfill specimen with the optimal mix proportion are analyzed by SEM–EDS and XRD. The results are shown in Fig. 9.

The XRD test results show that the main minerals in the backfill specimen with the optimal mix proportion are calcite (CaCO_3_), mullite, gypsum, ettringite (3CaO·Al_2_O_3_·3CaSO_4_·32H_2_O), quartz, dolomite (CaMg(CO_3_)_2_) and kaolinite, etc. The content of quartz, gypsum and calcite is relatively high. The broadened “convex closure” between 20◦ and 35◦ manifested weakly crystalline substances, such as amorphous C–S–H gel (calcium-silicate hydrate) which was generated from the alkaliactivated reaction of fly ash and the hydration of OPC^45^.

The micromorphology of the backfill specimen was observed by SEM. It was found that the internal structure of the specimen was loose and there were large voids. There were some CSW particles that did not participate in the hydration reaction, such as spherical CFA particles, indicating that there is no hydration reaction occurs between CSW particles. The surface of some spherical fly ash and its surroundings are wrapped with a thin layer of fluffy substances. EDS point scanning results show that the main elements in the area where the plush substance is located are Si, Mg, Al, Ca, O and a small amount of C, combined with the XRD test results, it can be seen that this area contains common minerals such as dolomite in fly ash, indicating that the X-rays penetrated through the thin layer of fluffy substances on the surface of fly ash during the EDS scanning process. It is comprehensively judged that the thin layer of fluffy substance is C–S–H gel, etc.

A large number of hexagonal prism-shaped product hydration product particles are attached to the interior and surrounding of the CSW particle voids. It is found by EDS point scanning that the main elements of the hexagonal prism-shaped product are O, Si, Al, Ca, S and C, and the molar fraction ratio of Ca and S is about 2, so the product is ettringite^46^, which is the hydration product of OPC and dihydrate gypsum in an alkaline water environment. Si and C elements were also detected in the EDS pattern, which indicated that part of Al_2_O_3_ was replaced by SiO_2_ and part of CaSO_4_ was replaced by CaCO_3_ during the hydration reaction^47,48^.

## Discussion

The large-scale disposal of multi-source CSW is increasingly becoming an urgent problem in the energy and chemical bases in western China. Considering the environmental impact, material composition and disposal scale, underground backfilling in coal mines is undoubtedly one of the most realistic solutions for large-scale disposal of multi-source CSW. As underground backfilling materials, multi-source CSW has multiple and complex components. It is necessary to adopt a material mix proportion scheme for accurate, economical and efficient underground backfilling in coal mines. The research on the mix proportion and microstructure of multi-source CSW materials has greatly reduced the blindness of large-scale synergistic backfilling with these solid wastes. For example, if the support performance of multi-source CSW backfill materials is improved, the amount of DG added can be appropriately reduced. This paper uses RSM-BBD and MDM to determine the mix proportion of CSW and cementitious materials with optimal comprehensive indicators. Finally, the microstructure of the backfill material with optimal mix proportion was studied. This study has obvious reference significance for engineering practice.

However, the optimal material mix proportion in this paper has only been tested at the laboratory scale. Although the performance of the multi-source CSW with optimal mix proportion is good, it has not been verified in the field application or semi-industrial practice (such as ring pipe experiments^13^, etc.). This is a limitation of this study and is also the future research work. In the future, multi-source CSW with optimal mix proportion can be carried out ring pipe experiments to test the transportation performance of the backfill materials, thereby providing feedback and improvement for the field application of backfill materials mix proportion.

The early strength of the multi-source CSW backfill material in this study is low, less than 2 MPa, and cannot meet the requirements of the mine backfill body to control roof subsidence and surface subsidence^14,49^. Low-strength backfill materials can only be used for grouting backfilling of underground cave spaces and bed separation spaces for large-scale CSW disposal, which greatly limits the application scope of backfill materials. Therefore, the next step of research focuses on improving the performance of backfill materials. By exploring the addition of other low-cost cementitious materials into multi-source CSW, the backfill material has certain fluidity and transportation properties while also rapidly solidifying and strengthening to support the overlying rock in a timely manner^50,51^.

After multi-source CSW is filled into underground space, it will be affected by groundwater soaking and leaching, and the environmental safety and stability of the backfill materials are unknown. Although some studies have shown that the toxicity and heavy metal leaching content of multi-source CSW are very low, and the pollution risk to the environment is controllable^52^, this depends on the material composition and source of multi-source CSW, and the environmental impact of large-scale underground backfilling with multi-source CSW are still unclear^53^. In this study, there is a lack of mine environmental risk assessment of multi-source CSW before and after filling underground space. This is also a research content to be carried out in the future. By testing, monitoring and evaluating the toxicity and pollution risks of multi-source CSW backfill materials at the laboratory scale and engineering scale, it provides scientific basis for the preparation of safe, green and environmentally friendly backfill materials.

## Conclusions

The optimal mixing proportions of DG, FBS and GFS determined by the RSM are DG/CG = 0.2, FBS/CG = 0.1 and GFS/CG = 0.1 respectively. The MDM was used to determine the mix proportion of backfill material with optimal comprehensive indicators as CG: CFA: DG: FBS: GFS = 1:0.5:0.2:0.1:0.1, the mass concentration is 78%, and cement accounts for 7.5% of the total CSW. The research results reduce the blindness of underground backfilling with multi-source CSW and provide a reference for engineering applications.

During the heating process, the backfill specimen showed a continuous weight loss phenomenon, which was caused by the evaporation of crystal water, structural water and hydroxyl water. The weight loss of the backfill specimen is about 26% in total at 800 °C. There are dense narrow-necked pores in the backfill specimen, and the pore connectivity is poor. The pore diameter range is 4–4 × 10^5^ nm, with mainly medium and large pores. The contents of quartz, gypsum and calcite in the backfill specimen are relatively high. There is no hydration reaction occurs between CSW particles, and the strength increase of the backfill material mainly depends on the cement hydration reaction products C–S–H gel and ettringite. In ettringite, part of Al_2_O_3_ is replaced by SiO_2_, and part of CaSO_4_ is replaced by CaCO_3_.

## Data Availability

The datasets used and/or analyzed during the current study are available from the corresponding author on reasonable request.
